# The influence of anaesthesia on cancer growth

**DOI:** 10.2478/raon-2024-0031

**Published:** 2024-06-11

**Authors:** Muhammet Selman Söğüt

**Affiliations:** Koç University Hospital, Istanbul/Turkey

Publish: March 1^st^, 2024.

Radiol Oncol 2024; 58(1): 9-14.

doi: 10.2478/raon-2024-0012

## To the editor

I read with a great interest the review recently published in Radiology and Oncology by Potocnik et al.^1^ Upon examination, I have identified a critical discrepancy between the review’s main text and the cited meta-analysis^2^ results, which seems to have led to a significant misunderstanding in the presentation of the findings.

The review states that in a recent meta-analysis^2^, patients with breast, esophageal, or non-small cell lung cancer had improved recurrence-free survival after receiving volatile anesthesia (VA) and that overall survival was longer after VA than after total intravenous anesthesia (TIVA). This statement contradicts the findings of the cited meta-analysis, which actually shows that TIVA is associated with improved outcomes both in terms of recurrence-free survival (pooled Hazard Ratio [HR], 0.78; 95% Confidence Interval [CI], 0.65 to 0.94; P < 0.01) and overall survival (pooled HR, 0.76; 95% CI, 0.63 to 0.92; P < 0.01) across several cancer types, including breast, esophageal, colorectal, gastric, and non-small cell lung cancer.

Interestingly, the conclusion section of the review correctly highlights the potential anti-inflammatory, antioxidant, and possibly antitumor effects of propofol (a common TIVA agent) compared to the proinflammatory effects of volatile anesthetics, which could accelerate metastasis. This conclusion aligns with the meta-analysis findings that favor TIVA over VA, suggesting a potential oversight or error in the review’s main text.

Given the significance of these findings for clinical practice and the potential impact on patient care, I believe a clarification and correction of the discrepancy in the review’s main text is crucial. Accurate representation of the meta-analysis results is essential for guiding future research and clinical decisions regarding anesthesia choice in cancer surgery.


**Notes**
No potential conflict of interest relevant to this letter was report.

References1.PotocnikIKerin-PovsicMMarkovic-BozicJThe influence of anaesthetic technique on cancer growthRadiol Oncol2024581914Available at: https://www.radioloncol.com/index.php/ro/article/view/42103837802710.2478/raon-2024-0012PMC108787702.YapALopez-OlivoMADubowitzJHillerJRiedelBGlobal OncoAnesthesia Research Collaboration GroupAnesthetic technique and cancer outcomes: a meta-analysis of total intravenous versus volatile anesthesiaCan J Anaesth2019665466110.1007/s12630-019-01330-x30834506

## Responses

### The authors reply

While reviewing the article^1^, we realised that we had made a mistake. Instead of VIMA, we should have written TIVA. Please, accept our apology. In the article, we also cited studies that concluded that volatile anaesthetics have anti-inflammatory action and so might act anti carcinogenic.^2,3,4,5,6^ In the conclusion we also wrote that this area is still quite unexplored and that studies have led to very controversial results. Regarding that please, find enclosed an additional reference of Wang J *et al.*^7^, who proved that volatile anaesthetics have a rule in the anti-cancer relevant signalling. Therefore, above mentioned mistake luckily did not have an effect on the message of the article.

**Assist. Prof. Iztok Potocnik, M.D., Ph.D.**
Institute of Oncology Ljubljana, Ljubljana, Slovenia
E-mail: vpotocnik@onko-i.si

**Prof. Jasmina Markovic-Bozic, M.D., Ph.D.**
University Clinical Centre Ljubljana, Ljubljana, Slovenia
E-mail: jasmina.markovicbozic@mf.uni-lj.si

**Notes**
No potential conflict of interest relevant to this letter was report.

References1.PotocnikIKerin-PovsicMMarkovic-BozicJThe influence of anaesthetic technique on cancer growthRadiol Oncol2024581914Available at: https://www.radioloncol.com/index.php/ro/article/view/42103837802710.2478/raon-2024-0012PMC108787702.El AzabSRRosseelPMDe LangeJJvan WijkEMvan StrikRSchefferGJEffect of VIMA with sevoflurane versus TIVA with propofol or midazolamsufentanil on the cytokine response during CABG surgeryEur J Anaesthesiol2002192768210.1017/s026502150200044341120744173.MinouAFDzyadzkoAMShcherbaAERummoOOThe influence of pharmacological preconditioning with sevoflurane on incidence of early allograft dysfunction in liver transplant recipientsAnesthesiol Res Pract2012201293048710.1155/2012/9304874222924040PMC34240504.JerinAPozar-LukanovicNSojarVStanisavljevicDPaver-ErzenVOsredkarJBalance of pro- and anti-inflammatory cytokines in liver surgeryClin Chem Lab Med20034189990310.1515/CCLM.2003.13643129405155.JabaudonMZhaiRBlondonnetRBondaWLMInhaled sedation in the intensive care unitAnaesth Crit Care Pain Med20224110113310.1016/j.accpm.2022.10113344359075986.SongZTanJEffects of anesthesia and anesthetic techniques on metastasis of lung cancers: a narrative reviewCancer Manag Res20221418920410.2147/CMAR.S3437724535046726PMC87635737.OhCSParkHJPiaoLSohnKMKohSEHwangDYExpression profiles of immune cells after propofol or sevoflurane anesthesia for colorectal cancer surgery: a prospective double-blind randomized trialAnesthesiology20221364485810.1097/ALN.0000000000004119350512638.WangJChengCSLuYSunSHuangSVolatile anesthetics regulate anti-cancer relevant signallingFront Oncol2021261161051410.3389/fonc.2021.61051433718164PMC7952859

## References

[1] Potocnik I, Kerin-Povsic M, Markovic-Bozic J (2024). The influence of anaesthetic technique on cancer growth. Radiol Oncol.

[2] Yap A, Lopez-Olivo MA, Dubowitz J, Hiller J, Riedel B, Global OncoAnesthesia Research Collaboration Group (2019). Anesthetic technique and cancer outcomes: a meta-analysis of total intravenous versus volatile anesthesia. Can J Anaesth.

